# 3D structure of the Campi Flegrei caldera central sector reconstructed through short-period magnetotelluric imaging

**DOI:** 10.1038/s41598-022-24998-6

**Published:** 2022-12-02

**Authors:** A. Troiano, M. G. Di Giuseppe, R. Isaia

**Affiliations:** 1 Sezione di Napoli ‘Osservatorio Vesuviano’, Istituto Nazionale di Geofisica e Vulcanologia, Naples, Italy; 2 Istituto di Geologia Ambientale e Geoingegneria, Consiglio Nazionale delle Ricerche, Roma, Italy

**Keywords:** Geophysics, Volcanology

## Abstract

The Campi Flegrei caldera experienced an unrest phase dating to 2005, which primary expression is the impressive ground uplift, accompanied by increasing degassing and seismic activities. Such last two phenomena developed mainly in the caldera central sector, including the Solfatara–Pisciarelli complex. However, the inner structure of such an area is still not defined, and this originates a poor understanding of the ongoing unrest. This paper describes the results of a new magnetotelluric survey performed in the Campi Flegrei caldera central sector. Through the inversion of data collected in 47 independent soundings, a 3D model of the electrical resistivity has been retrieved, which evidenced a partition of the investigated structure. The Agnano–Astroni area seems to be associated with a liquid-dominated geothermal reservoir, whereas the Solfatara–Pisciarelli area seems to be characterized by a single mixed liquid and gasses-dominated geothermal reservoir, which supplies the main caldera fumaroles. The proposed reconstruction of the geometrical characteristics of the hydrothermal system and the primary fluid rising pathways gives substantial clues about the significance of the detected structures in the evolution of the caldera unrest.

## Introduction

**Figure 1:**
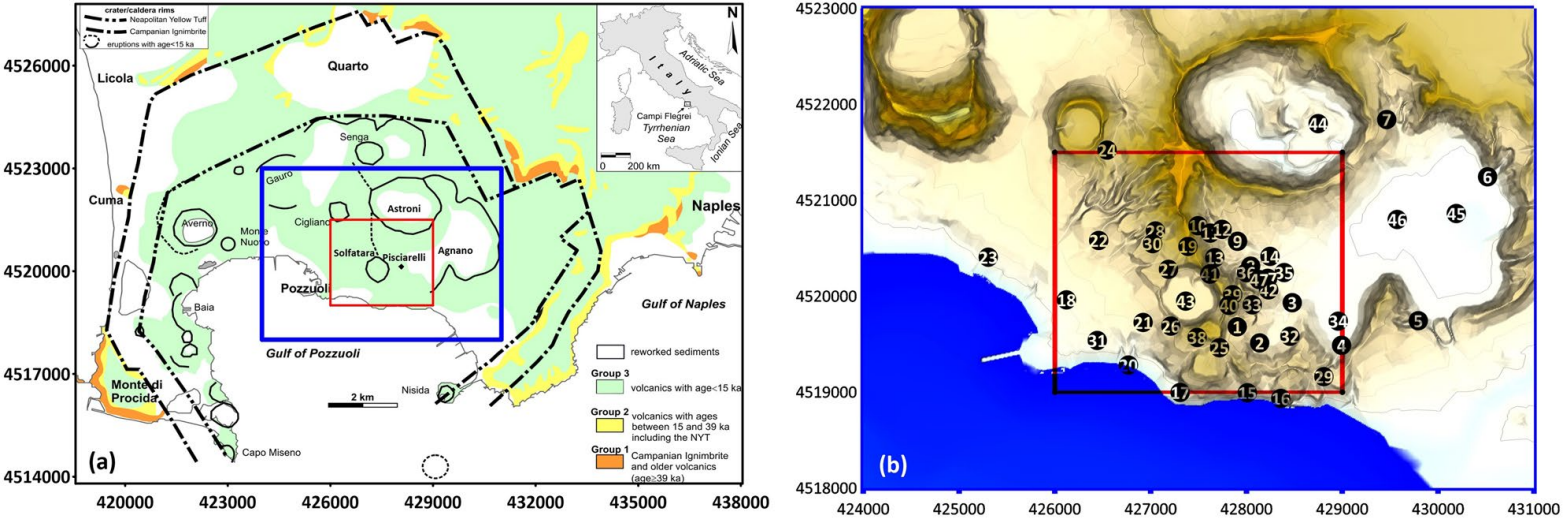
(**a**) Map of the Campi Flegrei caldera. Black lines indicate the main morphostructural features of the caldera. The area framed in the blue box has been magnified in panel (**b**); the red box indicates the investigated area (**b**) Map of the area subject of the MT surveying. The red square frame the investigated area. Black circles indicate the location of the MT sites.

The Campi Flegrei caldera (hereafter referred to as the CFc; Fig. 1) is one of the most dangerous volcanoes in Europe^1,2^. Intense and recurrent volcanism culminated in two main caldera-forming events, namely, the eruption of the Campanian Ignimbrite (39.8 ka) and that of the Neapolitan Yellow Tuff (15 ka)^3,4^, which marks the CFc eruptive history. After this last event, periods of quiescence separated at least 70 eruptions, mainly of explosive nature and small-medium magnitude, apart from a few more significant events that generated minor caldera collapses, such as the Agnano-Monte Spina (4.5 ka)^5^. The last eruption occurred in 1538 CE, leading to the formation of Monte Nuovo^6^. More recently, the CFc has been reactivated since 1950 CE, showing different unrest phases characterized by the alternation of ground uplift and subsidence^7^. The most relevant crisis occurred in 1982–84 CE, resulting in a 1.86 m uplift, with a maximum uplift rate of approximately one m/y, accompanied by low-magnitude seismic swarms (M_dmax_ = 4.0). The subsequent twenty years were a subsidence period that permitted a partial recovery of the ground uplift of approximately 1 m. However, the ground uplift started again in 2005, reaching about 90 cm since 2011, with a maximum uplift rate of 15.6 cm/year (in December 2022). Regarding seismic activity, the number of events/month has increased since 2018^7^. Moreover, after decades of very low seismicity (< 3 M_d_), among the 2492 seismic events recorded during the last twelve months, the 3.6 M_d_ earthquake of 29 March 2022 represents the most energetic earthquake since 1985.

Many literature papers fed the debate about the phenomena that characterize this last unrest phase. However, contrasting contributions link the CFc unrest to the effects of magma intrusion at shallow depths, exclusively to hydrothermal effects, or even to mixed magmatic-hydrothermal effects capable of producing unrest episodes (see Troise et al.^8^ and references therein). A definitive solution to such an issue is still missing, despite the different experimental observations carried out in the framework of regular CFc monitoring during recent decades.

In this framework, it is helpful to highlight a few characteristics of the recent CFc unrest. Regular monitoring of the active structures of the CFc evidenced anomalous gaseous emissions in many parts of the caldera^9^. The Pisciarelli zone represents the area of maximum emissions, located at approximately 500 m E to the Solfatara crater^10,11^. As in the case of the 1982–1984 unrest crises, a permanent radial pattern characterizes the observed ground deformation. According to literature evidence, the maximum ground deformation remains centered at approximately 2 km WSW to the Solfatara, where the ground level remains almost undisturbed^7^. In contrast, the seismogenic volume of the post-2000 activity seems to manifest an eastward shift from the 1982–1984 earthquakes^12^.

In summary, gas emissions and seismicity seem to be distal manifestations of the current caldera unrest, likely related to deep fluid injection of presumable magmatic origin. However, any suggestion of physical mechanisms could yet explain such dynamics. Without such knowledge, evaluating the level of possible volcanic hazard linked to the recent CFc unrest could be poorly constrained. This lack of knowledge calls for strengthening the structural definition of the CFc setting, intending to ascertain more data to help understand this aspect of the CFc volcanic activity. The present paper focuses on the Campi Flegrei central sector (hereafter referred to as the 'CFc-CS'), where, as previously mentioned, some of the most relevant volcanic reawakening signs manifest.

The CFc-CS is one of the most studied recent volcanic systems due to the presence of the Solfatara crater and the adjacent Pisciarelli fumarolic field. These latter are, at present, the most active caldera structures in terms of degassing and seismic activity^10,11,13–16^.

In recent years, literature papers have presented many geophysical results devoted to the structures of this sector of the caldera^1,15,17–21^. Among the various types of prospections, those based on electrical resistivity have already emerged as some of the most proficient sources of information aimed at understanding the role and distribution of geothermal fluids, their interactions with meteoric recharge, and the main structural lineaments, together with the effects of their circulation on the surrounding rock volumes (e.g., Isaia et al.^16^). Furthermore, electrical resistivity is highly sensitive to the presence of conductive fluids in the rock matrix. Consequently, the exploration of volcanically active areas often adopts resistivity-based techniques. During the last decade, prospections based on electrical resistivity tomography^12,13,22–25^, magnetotelluric^26,27^, and more recently induced polarization^11^ reconstructed the CFc subsoil at various depths in a proficient and non-invasive way.

However, additional geophysical images would still be valuable to ascertain the nature and geometry of the volcano-tectonic caldera structures. At the same time, the caldera hydrothermal system's configuration still has to be detailed with sufficient resolution. A significant improvement in magnetotelluric survey in the CFc area could likely contribute to overcoming such a knowledge gap. Such a technique is widely employed to characterize hydrothermal systems for both the exploitation of geothermal resources^28^ and the understanding of volcanic system purposes^29–32^ due to its unmatched capability to explore deeply buried structures through the reconstruction of the electrical resistivity contrasts in the subsoil, even at very great depths^33,34^. The same CFc-CS structures, especially Solfatara and Pisciarelli, have already been the object of geophysical investigations developed via such methodology during the last decade^26,27^. However, all these contributions involved 2D applications, and a proper 3D survey, which now represents the state-of-the-art magnetotelluric prospections, has not yet been applied to CFc-CS imaging.

A new 3D survey overcomes the limitations of the past magnetotelluric investigations carried out in the CFc-CS. Our results furnish an image of the red-framed area of Fig. 1a, which reconstruct the electrical resistivity in the first 2.5 km of depth through the acquisition of electromagnetic data in the 47 measurement sites indicated in Fig. 1b with black dots. The resulting 3D resistivity model unveils multiple findings about the CFc-CS that strongly enforce the current state of knowledge regarding the relationship between the Solfatara and the Pisciarelli feeding systems and the neighboring structures of the CFc-CS, such as the Astroni crater and Agnano plain.

## 3D resistivity model

The presented electrical resistivity model is the first three-dimensional reconstruction of the CFc-CS obtained via MT surveying. In the last few years, various authors developed 2D applications of such technique along alignments crossing the Solfatara crater, Pisciarelli fumarolic field, and Agnano plain^26,27^. The resulting 2D resistivity cross-sections highly contributed to the development of the Literature about the CFc, and several papers based their considerations on these MT studies^35,36^. However, the data dimensional analysis performed during these studies indicated a prevailing 3D character of the local structures. Consequently, the former 2D resistivity cross-sections could easily not fully match the actual character of the CFc-CS structures. The three-dimensional modeling presented here avoid such possible distortions while clarifying the significance of the principal anomalies detected with MT imaging. Note also that for a structure as complex and heterogeneous as the CFc, with multiple eruptive centers, the adoption of 3D modeling seems the most reasonable option to obtain a good understanding of the true significance of the detected anomaly.

**Figure 2:**
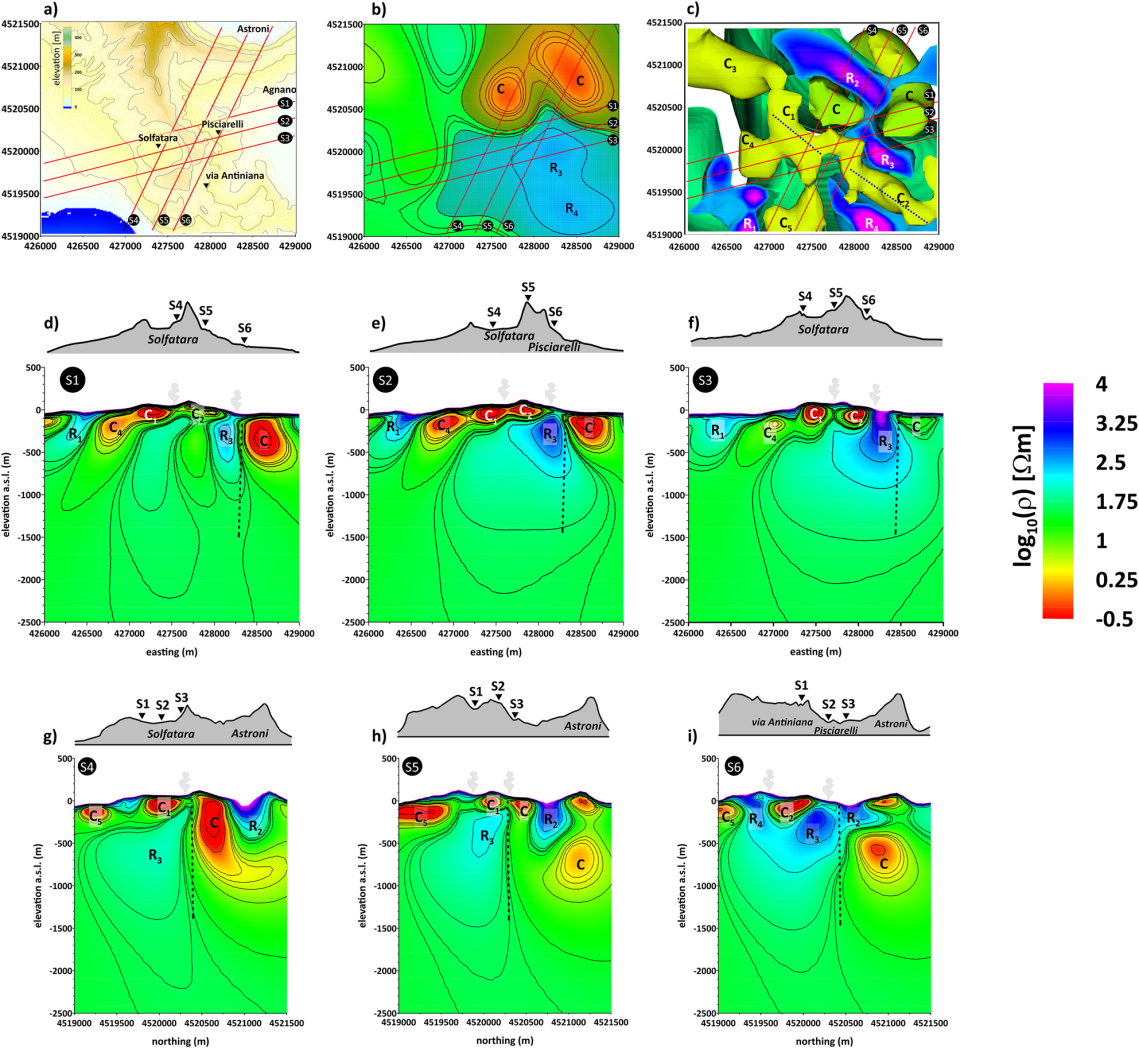
(**a**) Map of the area investigated through the MT surveying (**b**) plane map of the electrical resistivity at 700 m depth b.s.l. (**c**) isosurface representation of the 3D resistivity model in top view. The red lines in the last three panels indicate the directions of the S1–S6 profiles discussed in the text. (**d–i**): Resistivity cross-sections extracted by the 3D model along the S1–S6 profiles. The elevations along the profiles are reported along the top of the images. The main electrical features are labelled with capital letters. The black dotted lines indicate the transition between the S-PHS and the A-AHS (see text). The same common logarithmic scale represents the electrical resistivity in all the panels.

To gain insight into the 3D model, Fig. 2 displays six 2D resistivity cross-sections corresponding to the directions indicated by the solid red lines in Fig. 2a–c. Sections S1, S2, and S3, reported in Fig. 2d–f, respectively, develop along with the directions SW-NE, whereas sections S4, S5, and S6, reported in Fig. 2g–i, respectively, develop roughly in the N-S direction. In addition, Fig. 2b displays a resistivity map at 700 m b.s.l., and Fig. 2c shows an isosurface representation of the 3D model in the top view. Capital letters indicate the main electromagnetic anomalies characterizing the resistivity model.

The most relevant feature of the 3D model is the sharp vertical transition, indicated by dashed black lines in Fig. 2d–i, which divides the investigated volume into two separate and contiguous sectors with different resistivity trends and develops down to around 1500 m depth b.s.l.

Southward of this transition, an environment with resistivity ranging between a few to thousands Ω m, is located in correspondence with the Solfatara crater, the Pisciarelli fumarolic field, and the Via Antiniana degassing area (Fig. 2a).

Relatively conductive (less than 20 Ω m) anomalies, labelled C1-C2-C4 characterize the shallower portion of this sector, forming a continuous and less than 500 m thick stratum, which approximately aligns at NW–SE (as indicated with the blue dashed line in the isosurface plot in Fig. 2c). This confined conductive layer lies on a region comprises between 200 and 1500 m depth b.s.l. corresponding to higher resistivity ranging between a few hundred to thousands Ω m. In the upper and easternmost portion of this zone, two high resistivity cores (> 10^3^ Ω m) labelled R3 and R4, respectively, develop. (Fig. 2d–i). The R3 and R4 anomalies directly connect with the ground surface in correspondence with the Pisciarelli fumarolic field and the via Antiniana area. In addition, R3 presents a bulge that creeps between the conductive anomalies C1 and C2 and rises to the surface in the Solfatara area. (Fig. 2e,f).

Northward of the vertical transition, a relatively more conductive environment corresponds with part of the Astroni–Agnano (Fig. 2). The presence of a conductive anomaly denoted C characterizes this environment. This latter extends to a depth of about 1000 m, resulting deeper than the other conductive anomalies detected in the southern part of the CFc-CS (see Fig. 2d–i). In the northernmost shallow part of this sector, anomaly R2is also located, reaching about 500 m depth b.s.l., characterized by a resistive response (more than several hundred Ω m).

The described parting between the southern, relatively more resistive environment and the northern, more conductive environment is evident in the resistivity map shown in Fig. 2b (areas filled with blue and red polygons, respectively). We believe that this distinction corresponds to the separation between the Solfatara and Pisciarelli hydrothermal system (hereafter referred to as "S-PHS") and the Agnano-Astroni hydrothermal system (hereafter referred to as "A-AHS"), which gives rise to two separate and contiguous structures.

## Discussion

**Figure 3:**
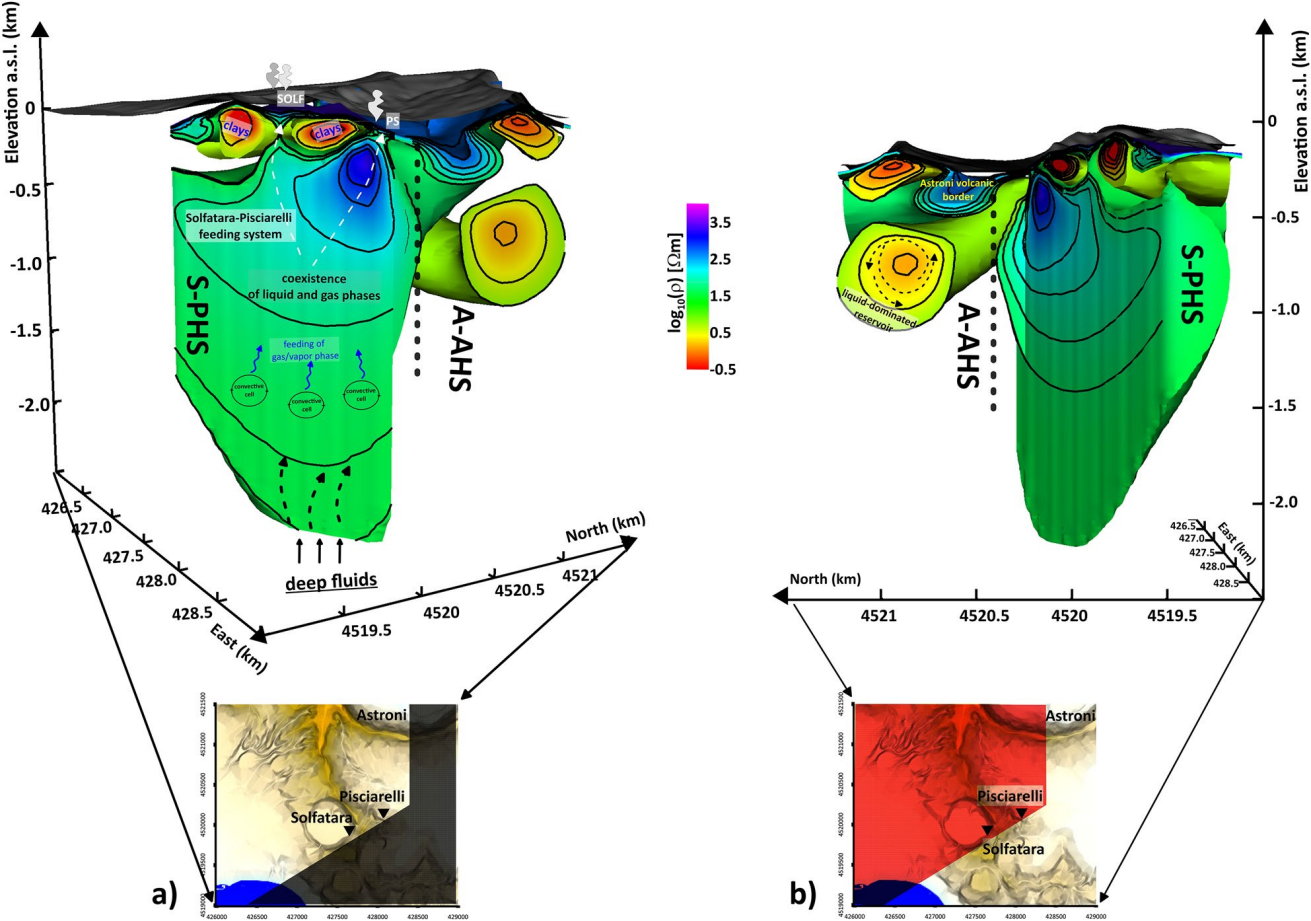
Sketch model of the CFc-CS deducted by the 3D resistivity model obtained through the current MT survey. Interpretative elements are also superimposed on the model. In particular, the location of the Pisciarelli (PS) and the Solfatara (BG and BN) fumaroles is indicated. The common logarithmic scale represents the electrical resistivity.

As mentioned in the previous section, the current MT imaging shows the division of the CFc-CS into two separate environments, the S-PHS and the A-AHS. Figure 3 sketches a 3D representation of these two contiguous volumes. Specifically, looking at the figure, the magnetotelluric picture of the S-PHS recalls the typical resistivity model for a geothermal reservoir in areas of high permeability and pervasive alteration^28^, where a mixture of fluids in the liquid and gaseous phases permeate the rock volume. In the last years, MT and Audio-MT applications have been increasingly concerned with 3D modelling of similar structures^29,32,37^, and often the results of such investigations show strong similarities to the model proposed for the S-PHS in the present study. Based on this Literature, the alternation of the blocks listed in the following well resumes the outlines evidenced in Fig. 3 for the structure of the S-PHS:

i. The uppermost conductive block of fewer than 500 m of thickness. This shallow structure appears continuous, except for a series of interruptions consisting of highly resistive bodies/anomalies connecting the underlying layers directly with the ground surface in correspondence with the sector's main emissive areas. (e.g., the Solfatara crater, Pisciarelli fumarole field and, via Antiniana). A few Ω m resistivities characterize the core of this shallower block. Recalling the discussion in Troiano et al.^27^, we note that in a volcanic-geothermal setting, a similar highly conductive body may indicate either a hydrothermally mineralized, clay-rich layer or a strongly hydrothermalized, water-bearing rock. Because clay minerals such as illite and smectite generally exhibit high conductivities in the presence of saline fluids^38,39^, we consider such a block to be a hot liquid-permeated structure whose circulation has triggered extensive hydrothermal alteration effects in a portion of the rock volume.
ii. The underlying resistive volume of about 1300 m of thickness. An electrical resistivity spanning from a few hundred up to several thousands of Ω m characterizes this block, which can be associated with the main geothermal reservoir of the S-PHS permeated by a mixture of fluids in coexisting liquid and vapor phases. The highly resistive structures before described, which interrupt the continuity of the shallower conductive stratum in correspondence of the Solfatara and Pisciarelli main fumaroles (Fig. 3), directly connect the geothermal reservoir to the ground surface. The most relevant among such resistive apophysis is the anomaly indicated as R3 in all the maps and sections of Fig. 2, which likely represents the trace of a gas plume feeding the Pisciarelli fumarole field. This anomaly was already detected in the 2D resistivity section of^27^ and at least in its shallower part by the 3D ERT survey by^12^. In contrast, a similar feature miss in the Solfatara-Pisciarelli-Agnano 2D resistivity profile performed by Siniscalchi et al.^26^.
iii. A deeper zone characterized by an electrical resistivity of tens of Ω m. This reduction in the resistivity could suggest an environment dominated by the liquid phase, with probably diffuse convective effects, which in turn strengthen the supply of vapors and gasses to the above-standing geothermal reservoir.

This setting suggests an afflux of hot fluids of likely magmatic origin and prevalently in the liquid phase into the S-PHS bottom. After ingression in the S-PHS, these hot fluid components rise upward due to the buoyancy effect, and, in this process, they form convective cells, which serve as a motor for energy transport between deeper structures and the surface. At a depth of about 1000 m b.s.l. the changes in the thermodynamic conditions (e. g. the lowering of the hydrostatic pressure profile and the high enthalpy proper of the deep-rising fluids) favor the vaporization processes that cause, at about 800 m b.s.l., the dominance of the vapor phase and the formation of the gas plume associated with the R3 resistivity anomaly.

The vapour-dominated part of the geothermal system extends to a depth of about 200 m b.s.l., where the clay formations associated with the shallower conductive anomalies confine the reservoir. However, as described in the previous section, two distinct apophyses imaged by the MT survey directly connect the reservoir with the ground surface. These structures are sensed as relatively resistive with respect to the surrounding highly conductive environment and represent two zones where fluids intrude between the clay formations overlying the geothermal reservoir. The most extended regions correspond to the Pisciarelli/Via Antiniana fumarole fields (Fig. 2d,e,f,i), the current principal emissive area in the caldera, cut by vast fault systems (Isaia et al.^16^). The second formation imaged as a narrow intrusion between the C1, and C2 anomalies (Figs. 2e,f and 3) corresponds with the large fumaroles (Bocca Grande/Bocca Nuova) within the Solfatara crater located along or at the crossing of faults^36^.

Then, the current MT image of the S-PHS structure suggests that a single deep geothermal reservoir fed the two structures of Solfatara and Pisciarelli, simultaneously reconstructing their shaping.

Such a result resolves the main unresolved questions regarding the relationship between these two structures, matching at the same time with many among the previous observations concerning the area.

The above-described MT imaging is consistent, in its shallower part, with the electrical resistivity imaged by the ERT (Electrical Resistivity Tomography) survey presented in Troiano et al. (2019). In particular, we note the correspondences between the R2 anomaly detected by the present MT survey and the resistive anomaly identified by ERT at the Astroni crater border, C1-C2 and the conductive structure corresponding to the Solfatara maar, R3 and the resistive anomaly at Pisciarelli.

The MT imaging is also consistent with the presence of a hydrothermal aquifer that, according to seismic observations, is located at a maximum depth of 2.5 km^40^. The shape of the S-PHS, as shown in Fig. 3, and the presence, as mentioned earlier, of an intense fluid component circulating in the pores and fractures of the rock are also consistent with the presence of a zone of very low rigidity, low velocity, and strong fracturing, where the fluids flowing through the fractures from the western part of the caldera are channelled and interact with the fluids and thermal components of magmatic origin coming from below^41^. The presence of porous rocks hosting a two-phase fluid mixture in a highly fractured volume is also consistent with a main gravity low, already interpreted by Young et al.^21^ as an image of a feeder system that includes aSolfatara-Pisciarelli structure. It is also worth recalling the findings of Di Giuseppe et al.^42^, where the clustering of a trivariate geophysical dataset allowed the reconstruction of a roughly horizontal interface of separations linked to layering within the S-PHS. This interface appears to be located at about 2 km depth b.s.l. below the Solfatara's main fumarole, rising to 1.5 km b.s.l. below Pisciarelli fumarole field. This shaping agrees with the S-PHS geothermal reservoir outline, shown by the model depicted in Fig. 3, particularly with the resistivity change that marks the transition between the liquid-dominated and the mixed liquid–vapour-dominated parts.

In the northern sector, the generally conductive environment associated with the A-AHS (Figs. 2b–i and 3) appears to be the likely result of the predominance of fluids in the liquid phase. As mentioned earlier, the boundary between S-PHS and A-AHS appears to be marked by an almost vertical sharp discontinuity, likely indicating the presence of a primary fault system. Recent literature papers highlighted the role of similar structures in controlling fluid patterns in the shallower CFc geothermal system^11,16^, stressing the relevance of major fault systems in the overall setting of the area along with the idea of their central role in the emplacement of the local hydrothermal system. Current MT imaging, with its ability to detect deep structures, is helping to capture the full extent of this structural type for the first time and may support the idea that a fault zone acts as a relevant permeability barrier to fluids due to mineral precipitations filling the pore spaces^43^. The fault gauge could likely act as an impermeable sheet, whereas the damage zones could likely act as zones of intense fracturing (e.g., extremely enhanced permeability). However, we think of different behaviours that differentiate this permeability barrier's two sides. On the Agnano-Astroni side, fluid circulation should be facilitated by the higher permeability, with a consequent lowering of the fluid pressure. On the other side, towards the Solfatara-Pisciarelli structure, also likely effects of mineral precipitations in the rock pores should be considered. Accordingly, the faults could impede the mixing of fluids between the A-AHS and the S-PHS, which could lead to less enrichment of the S-PHS with meteoric water, which in turn could contribute to determining the thermodynamic conditions inside that favour the presence of mixed liquid and vapour phases. At the same time, a high degree of fracturing in the damage zones enhances the fluid flow through the faults, which also act as guidelines. In this way, the faults recall the hot fluids, and this process favours the hydrothermal alteration of the rock and the formation of clay minerals in their interior. A similar mechanism likely rules the interrelationship between the main resistive/conductive transition and the shallow C1-C2-C4 conductive bodies.

This primary fault system also likely determines the configuration of the C conductive anomaly (Figs. 2 and 3), which corresponds with a shallower hydrothermal system characterized by fluids mainly in the liquid phase due to mixing with shallow meteoric water. This system seems to be fed by hydrothermal fluids comparable to the composition of the fluids released by the Solfatara fumaroles^44^ and has been detected in the same sector also by gravimetric survey^21,45^ below the Astroni volcanic edifice, here marked by the R2 resistive anomaly (Figs. 2g–i, 3).

## Conclusions

The CFc-CS plays a critical role in recent volcanic activity throughout the phlegrean region, particularly in the recent unrest that increased the alert level to yellow. The current MT imaging provides insight into the structural setting and configuration of the CFc-CS, being the 3D resistivity model able to distinguish the differences between the two parts of which the hydrothermal system seems to be composed, namely the A-AHS and the S-PHS. In correspondence with the A-AHS, a substantial reduction in resistivity probably relates to the strong circulation of hot geothermal water with high concentrations of dissolved salts acting as conductive electrolytes in the rock matrix. On the other side, the S-PHS consists of a more resistive environment, probably due to the presence of drier formations with reduced liquid circulation. This diversification includes structures that develop at great depths (more than 1 km) and likely relates to the presence of two different supply systems, each feeding one of these two parts of the CFc-CS, combined with the effects of an extensive fault system.

In the context of the ongoing caldera unrest, the many original elements revealed by the 3D resistivity model furnish new clues about the possible evolutive scenario. As well-known in the context of a volcano-geothermal system, the upwelling of hydrothermal fluids could likely trigger eruptive phenomena, primarily in the presence of a shallow impermeable cap layer, usually composed of clay minerals, which can allow the accumulation of pressurized steam or superheated water directly below the top of the geothermal system^46–52^. Consequently, the permeability along the path of the hydrothermal fluids, which closely correlates with patterns of electrical resistivity, essentially controls the probability of a shallow eruptive event. As described in the previous sections, the MT imaging detected a single, almost vertical geothermal system supplying both the Solfatara and Pisciarelli fumarole fields. A single vapor-dominated reservoir, of about 1300 m thickness and confided below the 200 m depth b.s.l., feeds both structures. Remarkably, two distinct apophyses rise from such a reservoir, sourcing the Solfatara and the Pisciarelli fumarolic fields, representing the most significant hydrothermal manifestations of the caldera at present. The accurate geometrical definition of the geothermal reservoir and the detection of the primary fluid rising pathways retrieved through the 3D resistivity model increase the knowledge about the most significant structures in the possible evolution of the caldera unrest. The 3D image allowed us to identify below the Pisciarelli sector a shallow resistive anomaly previously not detected by other MT surveys, which cannot be excluded deriving from the gas movement that occurred during the last 20 years of CF unrest. This could be the base for new studies, including numerical simulations, aimed to provide further information to enhance the understanding of the process involved in the dynamics of the caldera.

Particularly, the relevance of the link between the Solfatara and Pisciarelli structures and the unrest phenomena enhances the centrality of such an area in the possible scenario, including also eruptive hydrothermal/phreatic events.

## Methods

### Magnetotelluric dataset and preliminary analysis

Magnetotelluric (MT) is a broadband passive geophysical methodology that reconstructs the electrical resistivity spatial distribution by analyzing the synchronous fluctuations of the electric and magnetic fields naturally induced in the subsoil by external sources. MT is a frequency-based method that does not require any form of an artificial source: at a single survey point on the Earth's surface, both the spectral analysis of the electric and magnetic natural fields recorded during the same time windows enables the estimate of the four-component **Z** tensor, also called the impedance tensor, which relates **E** and **H** following the relationship $$\left( {\begin{array}{*{20}c} {{\varvec{E}}_{\varvec{x}} } \\ {{\varvec{E}}_{\varvec{y}} } \\ \end{array} } \right) = \left( {\begin{array}{*{20}c} {{\varvec{Z}}_{\varvec{xx}} } & {{\varvec{Z}}_{\varvec{xy}} } \\ {{\varvec{Z}}_{\varvec{yx}} } & {{\varvec{Z}}_{\varvec{yy}} } \\ \end{array} } \right)\left( {\begin{array}{*{20}c} {{\varvec{H}}_{\varvec{x}} } \\ {{\varvec{H}}_{{\varvec{y}}} } \\ \end{array} } \right)$$**.**

The **Z** tensor depends on the signal frequency and the physical properties of the medium. Its four components, usually separated into diagonal (Z_xx_ and Z_yy_) and nondiagonal (Z_xy_ and Z_yx_) modes, contain information on the electrical structure of the subsoil related to the behaviour of the curves of apparent resistivity (ρ_ij_) and phase (φ), defined as: $\rho_{{{\varvec{ij}}}} = \frac{1}{{\user2{\omega \mu }}}\left| {{\varvec{Z}}_{{{\varvec{ij}}}} } \right|^{2} ;$

$\user2{\varphi }_{{{\varvec{ij}}}} = {\varvec{arctan}}\left( {\frac{{{\varvec{Im}}\left( {{\varvec{Z}}_{{{\varvec{ij}}}} } \right)}}{{{\varvec{Re}}\left( {{\varvec{Z}}_{{{\varvec{ij}}}} } \right)}}} \right)\user2{ }$, where μ and *f* indicate the magnetic permeability and the frequency, respectively. A vast amount of Literature describes the basic principles of the MT and its practical aspects. We cite, among the others^33,34,53^.

Figure 1 shows the locations of the 47 sites where fieldwork and MT data collection have been performed. A Stratagem EH4 instrument produced by Geometrics Measurements performed themeasurements, equipped with additional low-frequency magnetometers and electric dipoles to acquire signals in the [10^−1^–10^5^] Hz frequency band. A controlled source was used in the frequency range from 1000 to 64,000 Hz, which consists of two perpendicular vertical loop antennas. Each antenna has a moment of 400 Am^2^. This transmitter setup provides polarized source fields, allowing for tensor measurements of the ground resistivity with an enhanced signal-to-noise ratio, even in the case of data collected in areas of high cultural noise. However, near the antennas, such a source field will likely consist of waves with a spherical front that will not be uniform. We recall that the depth of penetration of the electromagnetic waves into the ground could be estimated by adopting the well-known definition of δ ≈ 500(ρT)^1/2^, where δ is the skin depth in m, ρ is the ground resistivity in Ωm and T is the period in s^54,55^. Both experimental results and numerical simulations^54,56^ indicate that at distances greater than 3δ from the source transmitter, the uniform and plane portion of the waves is dominant. Starting from 6δ, the waves can be considered entirely uniform and plane relative to the precision with which one can measure them^57^. Then we use the results of ERT, previously carried out in the investigated area^12^, to establish the shortest transmitter–receiver distance, which was maintained between 3 and 6δ, preferably as close as possible to 6δ.

The data acquisition phase coincides with the total lockdown related to the COVID-19 emergence, a period of almost total suspension of civil activities in Italy. In particular, the severe limitation of local and national train circulation represents a reduction of the most relevant sources of coherent noise in the electromagnetic data (see the discussion in Di Giuseppe et al.^58^). Fig. SM01 of the supplementary materials reproduces a few examples of the collected MT curves. Using the commercial software MT-Corrector produced by Zond permitted the subsequent remotion of non-smooth responses, approximating the frequency dependence of the impedance tensor Z by a smoothing spline.

Preliminary analyses of the collected data, described in the supplementary materials, permitted the evaluation of the data dimensionality and the presence of effects related to the CFc topography and bathymetry. These analyses have been described in the Supplementary Materials.

### Magnetotelluric data inversion

As described in the previous section, the PT analysis shows that the structures belonging to the central sector of the CFc retain a symmetry that leads to the need for a full three-dimensional inversion of the data. We recall that a 1-D inversion accounts for only resistivity variations with depth at each MT station and may be inaccurate in more complex geological settings^31^. The 2D case, on the other hand, implies that currents flow essentially either parallel or perpendicular to the strike direction, and then only the nondiagonal modes of the Z tensor are significant. Imposing a similar approximation during the data inversion could easily introduce spurious structures into the resistivity section, as created by the adopted algorithm to justify the effects of out-of-line structures over the considered alignment. Unlike 1D and 2D algorithms, a 3D inversion does not require any assumptions about the subsurface resistivity distribution, and despite the higher computational costs, this approach can be considered the most adequate for geological settings as complex as the Solfatara-Pisciarelli complex. For this reason, the parallel version of the 3D code Modular system for Electromagnetic inversion (ModEM)^59–61^ has been employed for data inversion, which was run with full impedance tensor elements (Z_xx_, Z_xy_, Z_yx_ and Z_yy_).

A homogeneous half-space has been considered as starting and reference model. Several tests have been performed to select its electrical resistivity. During such tests, different values of the half-space electrical resistivity have been considered. Further, a model has been derived from the determinant of the impedance tensor. In conclusion, 100 Ω m has been selected, corresponding to the best final data fitting. In any case, the inversion seems to converge toward models that are quite similar in most cases.

Considering a large amount of data and model parameters, along with numerous settings that control the inversion algorithm, some care is required to obtain a satisfactory result^62,63^. In practice, a different resistivity model can be obtained from the inversion simply by choosing the data frequencies or sizes of the cells in the model, among other parameters. Therefore, many inversion settings and parameters should be tested to understand the consistency and reliability of the inversion results^31^. Ideally, the MT stations should be uniformly distributed over the entire investigated area, and when the subsoil is discretized, there should not be more than one MT station in each model cell. Moreover, a few model cells should be present between each pair of MT stations to ensure that the inversion can place resistivity variations between stations, mainly when small, near-surface features are present. A trade-off is present between including more data/model cells and incurring a higher computational cost. In the end, the model mesh adopted during the inversion of our MT dataset includes bathymetry and topography and contains 88 cells in the north–south direction, 60 in the east–west direction, and 70 in the vertical direction (plus 10 air layers). Layers above sea level have a thickness of 10 m, and the first layer below sea level has a thickness of 10 m that increases by a factor of 1.2 for each subsequent layer down to a depth of 3 km. The first layer below a depth of 3 km has a thickness of approximately 500 m, increasing by a factor of 1.4 for each subsequent layer. The core mesh contains 80 m by 80 m horizontal cells to allow room for multiple model cells between each station. The high-resolution bathymetry of the CFc-CS coastline was included as a priori information and kept fixed during the inversion. A resistivity value of 0.3 Ω m was used for the seawater. Model regularization employed a smoothing parameter of 0.4 for both horizontal directions, and 0.3 for the vertical direction.

The input data were decimated to 14 logarithmically spaced frequencies, ranging from 1000 to 0.56 Hz. The highest and lowest frequencies determine the smallest and largest resolvable depths. Each datum in the inversion must be assigned an error (uncertainty) estimate. This value is essential as the inversion seeks a solution to minimize the misfit of each datum. Significant error estimates may cause the inversion to fit the measured data inadequately; error estimates that are too small may cause noise to be fit and result in a rough model. The standard impedance errors obtained from time-series processing may be minimal compared to the impedance values (< 1 per cent), and it may be necessary to apply an error floor to obtain a satisfactory inversion result. In our inversion, we diversify the error floor applied to diagonal and nondiagonal modes, considering the estimation of the sea and topographical effects described in the previous section. We applied a fixed error floor equal to 5 per cent of |Z_ij_| on the nondiagonal modes and 10 per cent of sqrt(|Z_ij*_Z_ii_|) on the diagonal modes.

For the preferred inversion model, 97 interactions were required to fit the full impedance tensor. As a metric for the fit of the modelled to the observed data, the commonly used normalized root mean square has been adopted, which is defined as $nRMS = \sqrt {\left( {N_{d} - 1} \right)^{ - 1} \mathop \sum \limits_{j = 1}^{{N_{d} }} \left( {\frac{{d_{obs,j} - d_{calc,j} }}{{\sigma_{j} }}} \right)^{2} }$, where *d*_*obs,j*_ and *d*_*calc,j*_ are observed, and calculated data, respectively, and σ_*j*_ represents the errors for all *N*_*d*_ data points. A value of this metric close to 1 is commonly interpreted as a data fit within the range of observational error, that is, in the case of this study the error floors, which somewhat reduces the statistical significance^64^. Figure SM07 shows a map of the final nRMS, which achieved a value of about 1.7. The fit to the majority of observations can also be deduced from the fit of the data curves presented in the Supplementary Materials. The consistency of the main features of the preferred inversion model was questioned by performing a series of tests in order to analyze every significant structure separately, using the model perturbation method. In addition, the Kolmogorov–Smirnov test has been employed to evaluate if the anomalies are statistically 'detected' by the MT data. The results of the resolution tests have been summarized in Section S3 of the Supplementary Materials.

## Supplementary Information

Supplementary Information 1.

Supplementary Information 2.

Supplementary Information 3.

Supplementary Information 4.

Supplementary Information 5.

Supplementary Information 6.

Supplementary Information 7.

Supplementary Information 8.

Supplementary Information 9.

Supplementary Information 10.

Supplementary Information 11.

Supplementary Information 12.

## Data Availability

All data generated or analyzed during this study are included in this published article [and its supplementary information files].
