# Supplementary figures and images for: 3D structure of the Campi Flegrei caldera central sector reconstructed through short-period magnetotelluric imaging
Source: Sci Rep. 2022 Dec 2;12:20802. doi: 10.1038/s41598-022-24998-6 (PMC9716173; doi:10.1038/s41598-022-24998-6)

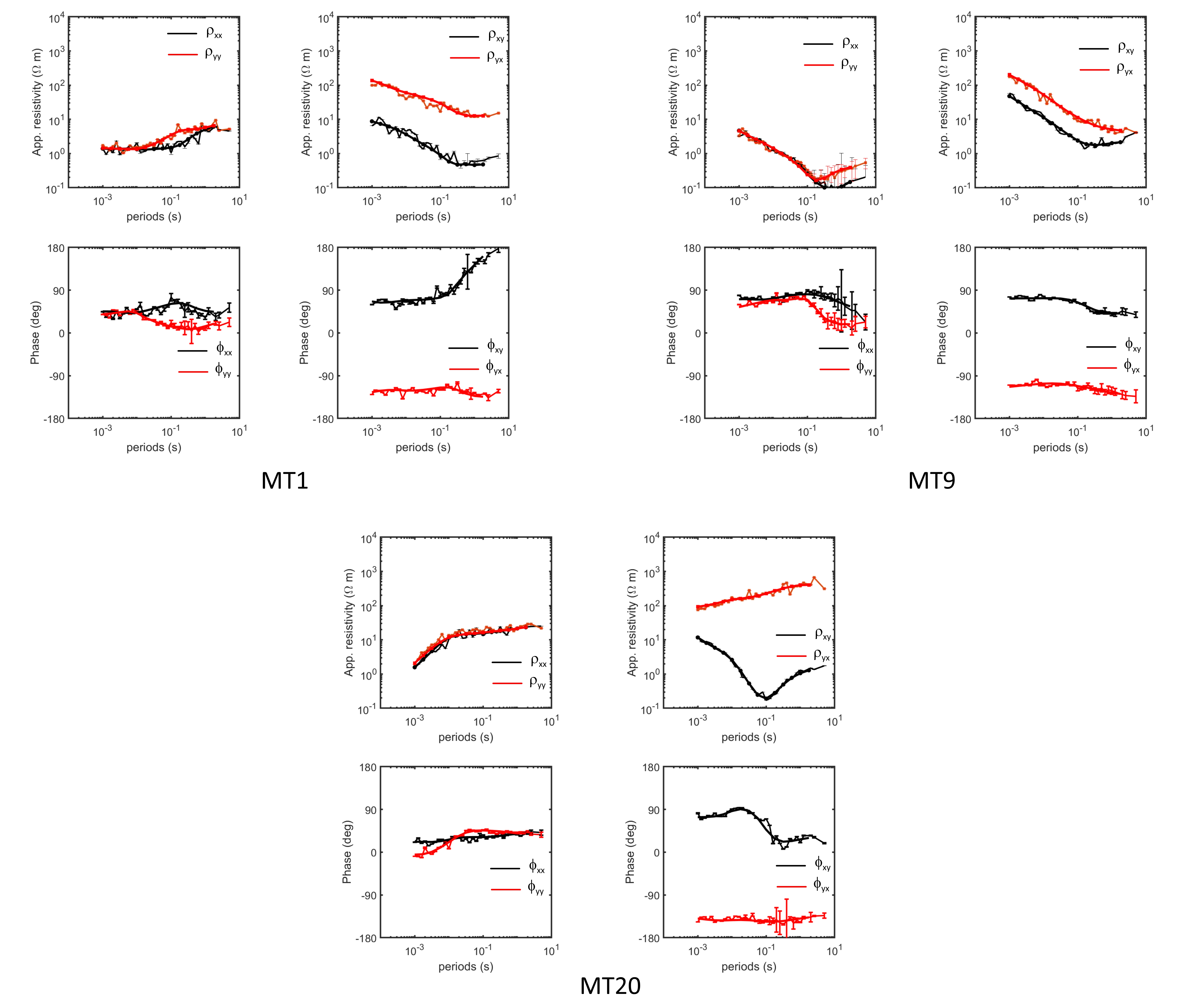

Supplement: Supplementary file 1 — Supplementary Information 1. [file 41598_2022_24998_MOESM1_ESM.jpg]

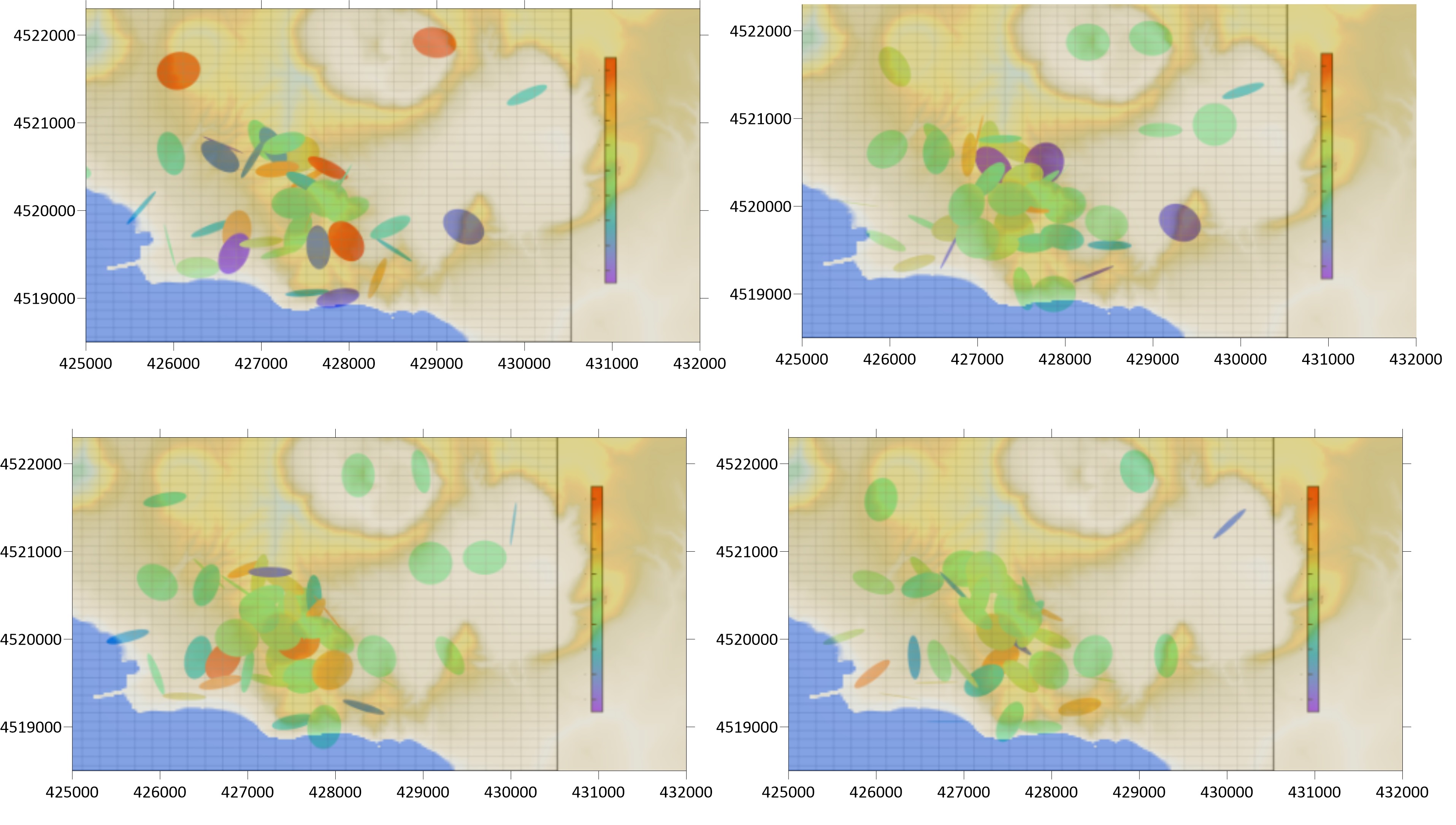

Supplement: Supplementary file 2 — Supplementary Information 2. [file 41598_2022_24998_MOESM2_ESM.jpg]

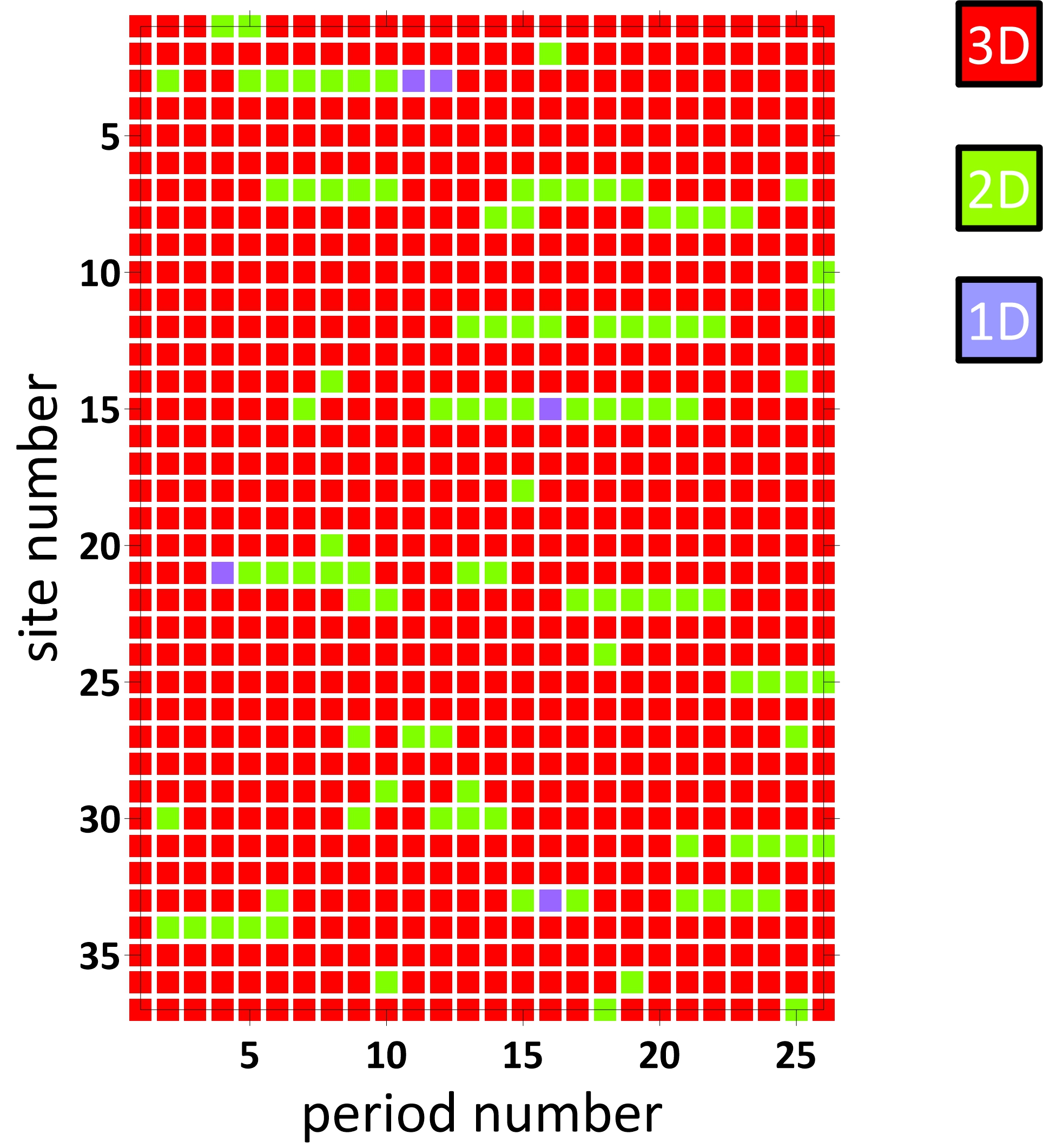

Supplement: Supplementary file 3 — Supplementary Information 3. [file 41598_2022_24998_MOESM3_ESM.jpg]

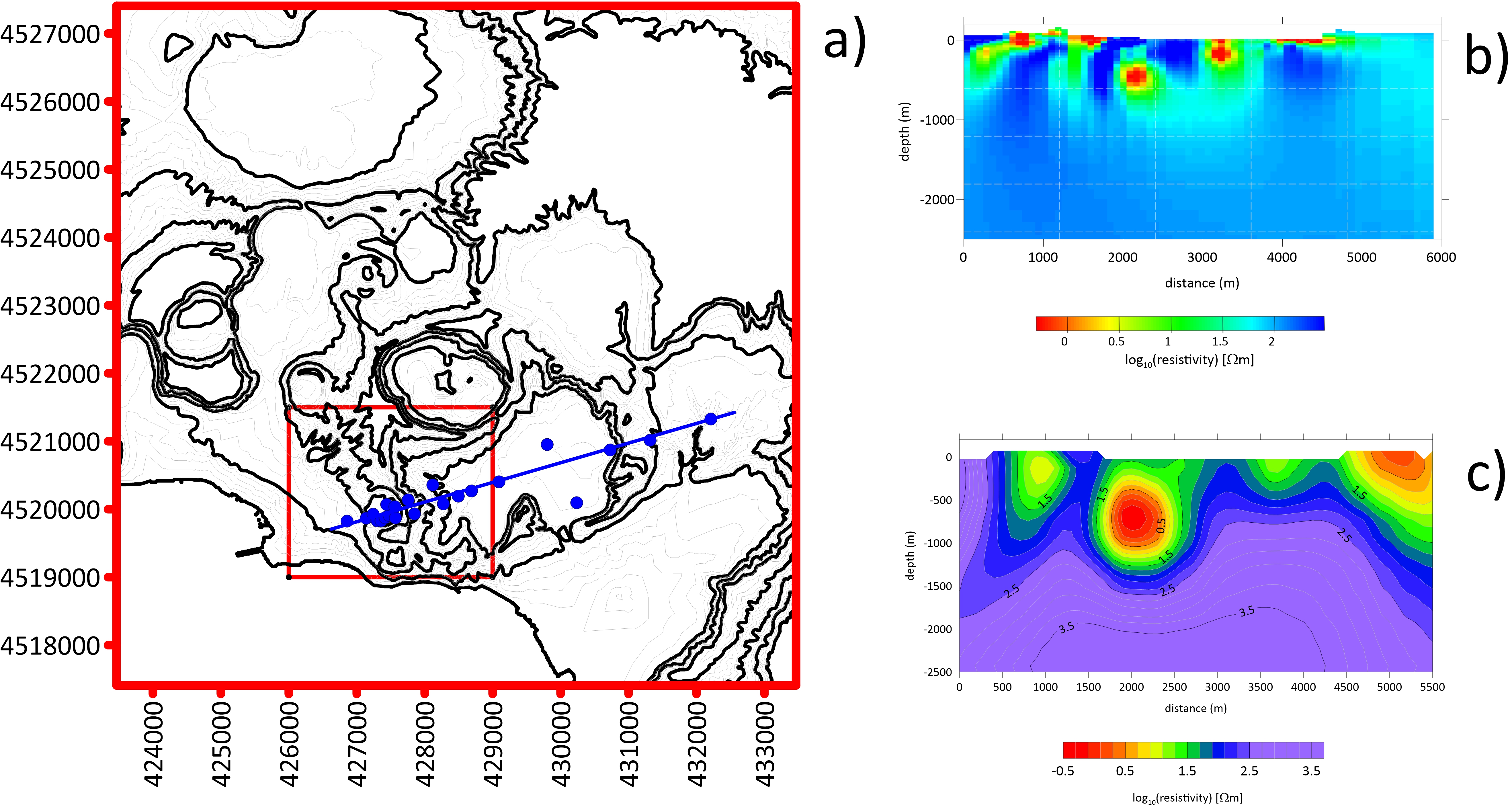

Supplement: Supplementary file 6 — Supplementary Information 6. [file 41598_2022_24998_MOESM6_ESM.jpg]

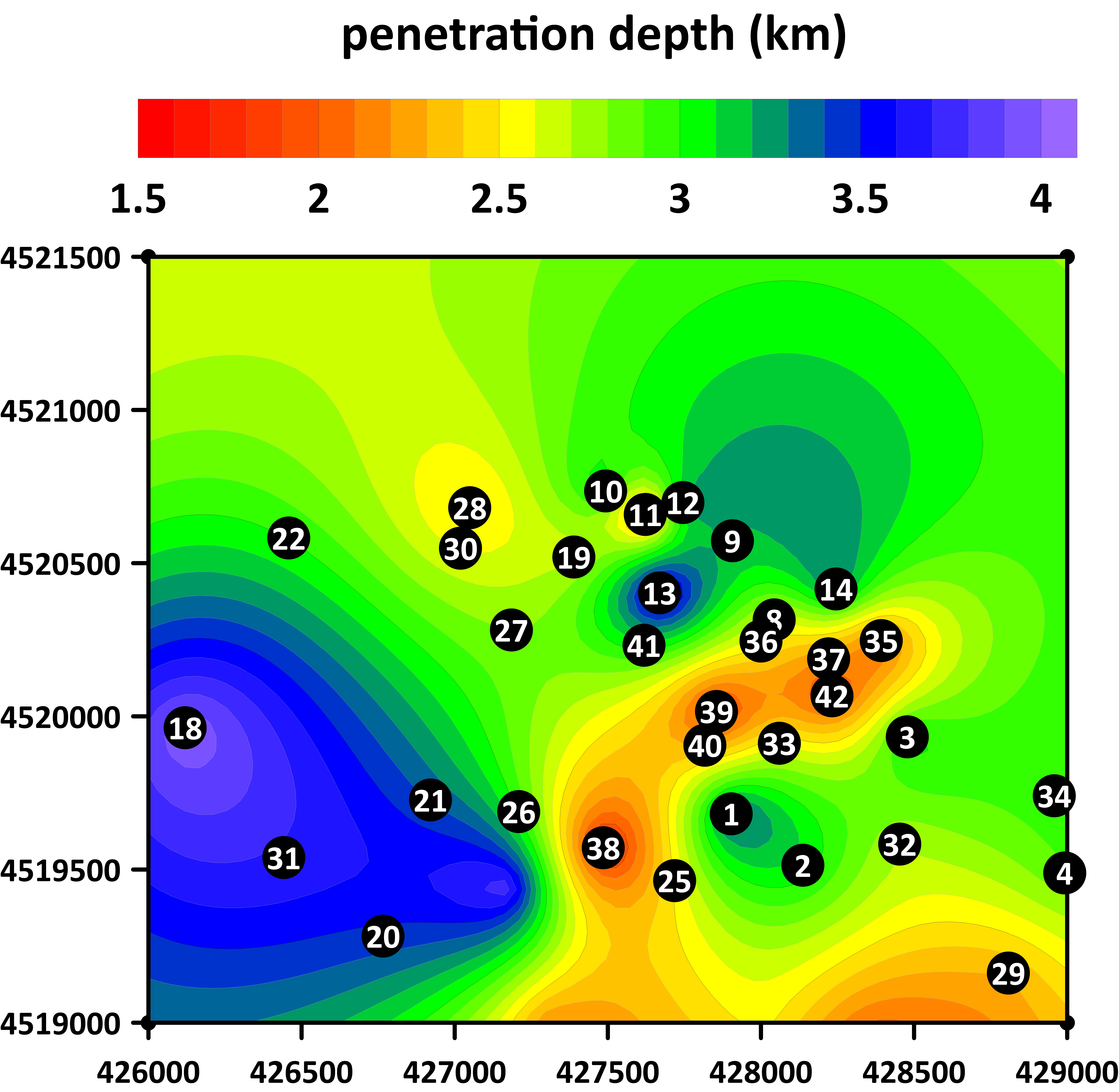

Supplement: Supplementary file 7 — Supplementary Information 7. [file 41598_2022_24998_MOESM7_ESM.jpg]

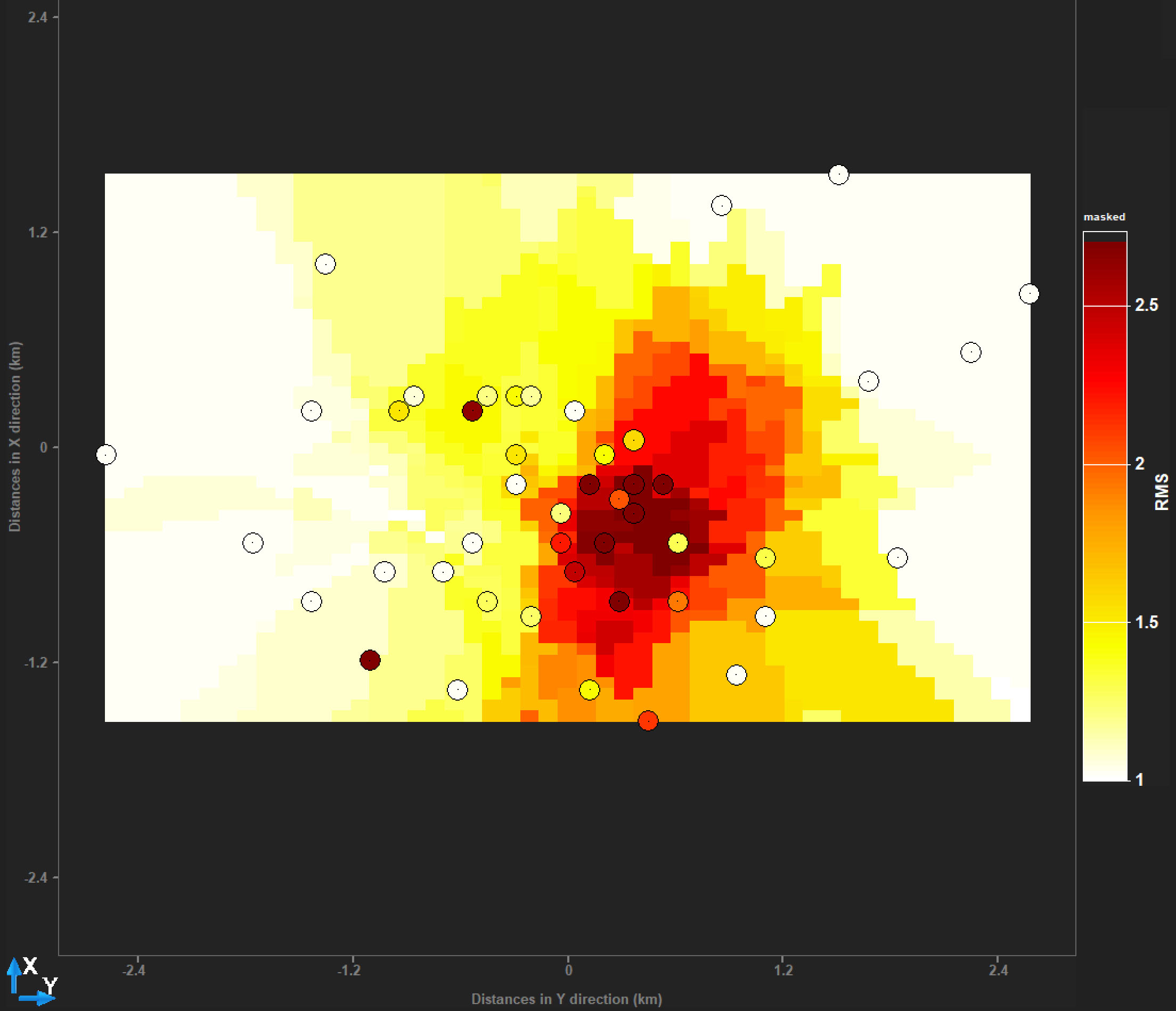

Supplement: Supplementary file 8 — Supplementary Information 8. [file 41598_2022_24998_MOESM8_ESM.jpg]

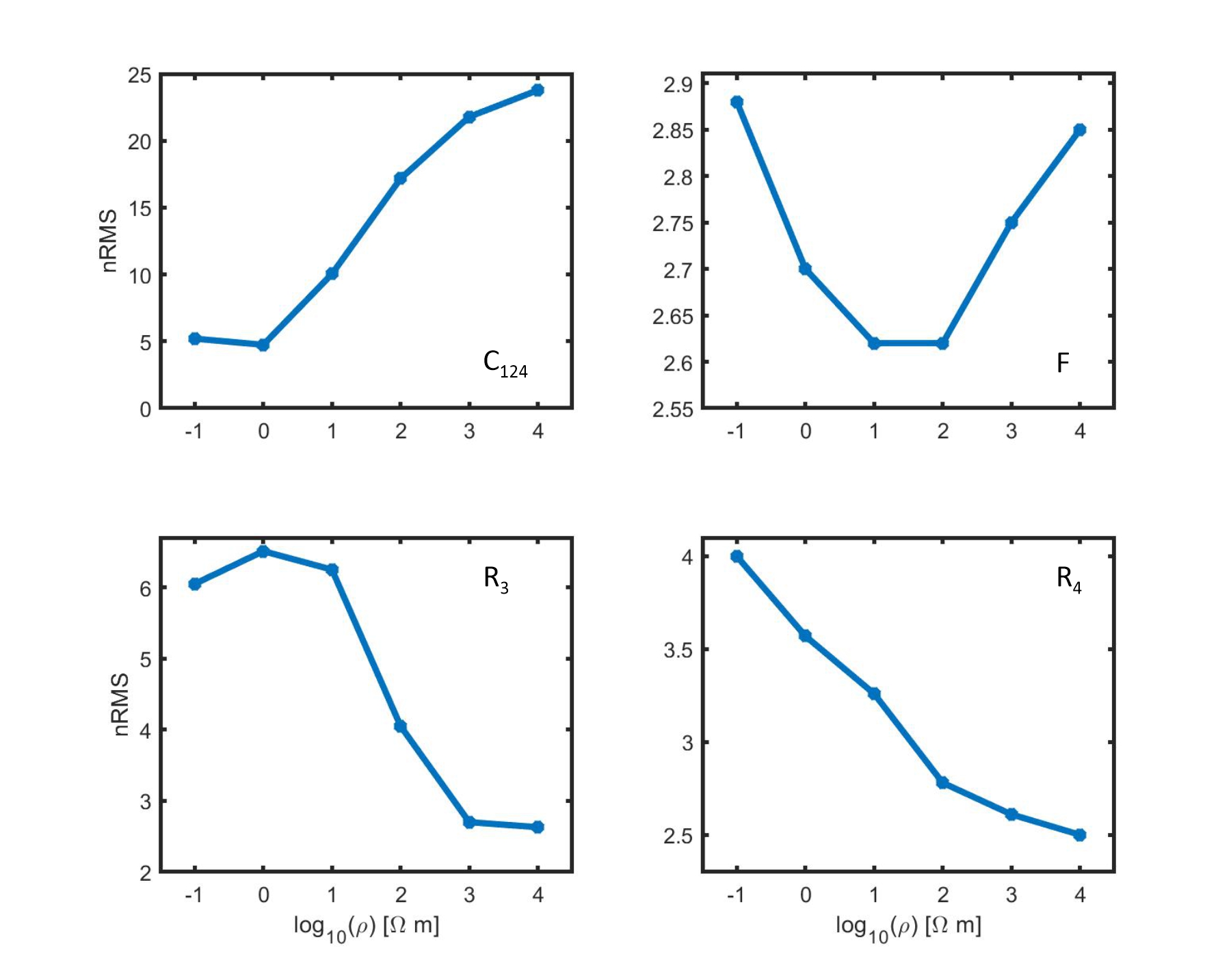

Supplement: Supplementary file 9 — Supplementary Information 9. [file 41598_2022_24998_MOESM9_ESM.jpg]

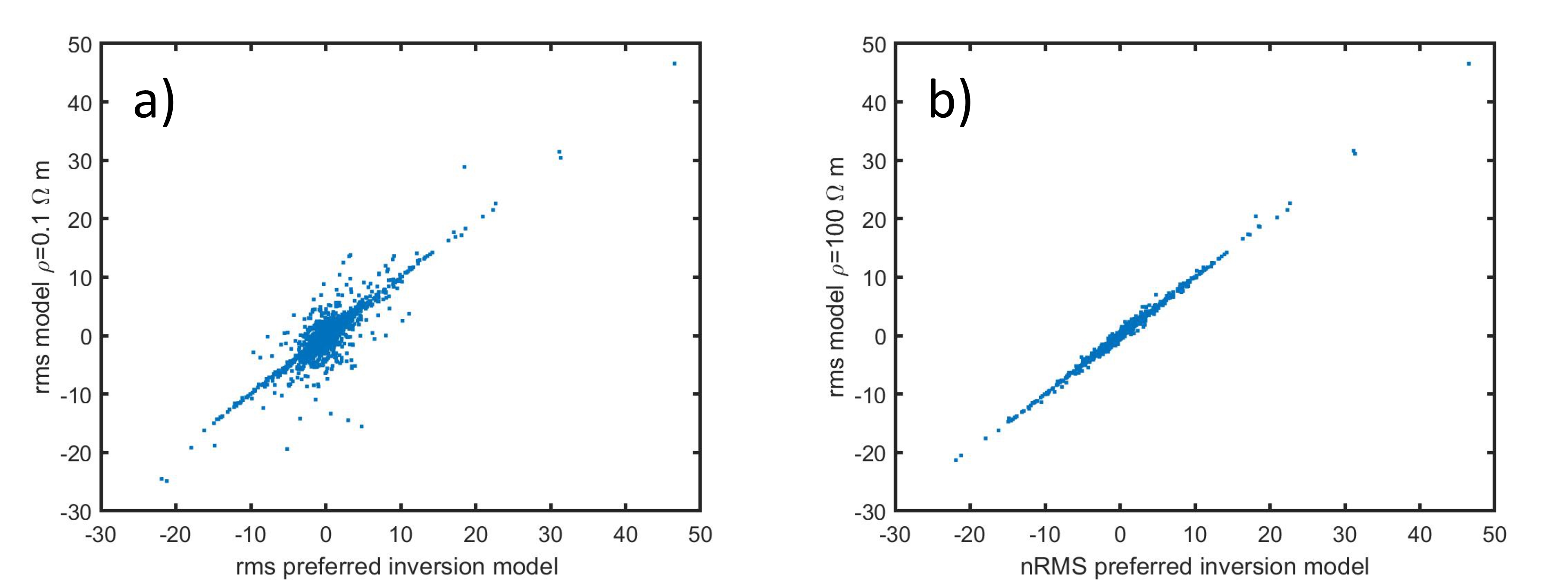

Supplement: Supplementary file 10 — Supplementary Information 10. [file 41598_2022_24998_MOESM10_ESM.jpg]
