# Supplementary material for: 3D structure of the Campi Flegrei caldera central sector reconstructed through short-period magnetotelluric imaging
Source: Sci Rep. 2022 Dec 2;12:20802. doi: 10.1038/s41598-022-24998-6 (PMC9716173; doi:10.1038/s41598-022-24998-6)
Supplement: Supplementary file 11 — Supplementary Information 11. [file 41598_2022_24998_MOESM11_ESM.docx]

*Scientific Reports*

Supporting Information for

**3D structure of the Campi Flegrei caldera central sector reconstructed through short-period magnetotelluric imaging.**

A. Troiano^(1,*)^, M. G. Di Giuseppe^(1)^, R. Isaia^(1)^.

1 Istituto Nazionale di Geofisica e Vulcanologia, Sezione di Napoli ‘Osservatorio Vesuviano’ – Italy.

**Contents of the supplementary materials.**

This Supporting Information file contains details on the MT prospection presented in the paper. Section S1 describes the preliminary analyses performed on the MT data to evaluate their dimensionality and the likely presence of effects related to the CFc topography/bathymetry. Section S2 describes the forward modelling aimed to evaluate the effects that 2D approximation could have on the MT data inversion. Section S3 describes the results of several tests concerning the resolution of the main anomalies detected by the preferred inversion model.

Concerning the supporting figures, Figure SM01 reproduces a few examples of the collected MT curves; Fig. SM02 shows the Phase Tensor polarization ellipses for periods of 0.001, 0.01 s, 0.1 s, and 1 s, respectively; Fig. SM03 resumes the results of data dimensional analysis for the MT dataset; Fig. SM04 resume the result of forward modelling aimed to evaluate the effects of the topography and the near coastlines on the MT data; Fig. SM05 show the results of forward modelling aimed to evaluate the possible effects of the use of 2D approximation on the collected MT data; Fig. SM06 shows a map of the estimated penetration depth of the electromagnetic waves of 3 s period; Fig. SM07 shows the results of a series of tests on the resolution of the most relevant resistivity anomalies present in the preferred inversion model; Fig. SM08 compares the residual relative to two edited models, where different resistivity values have been attributed to the bottom of the S-PHS. Fig. SM09 shows a map of the nRMS for the preferred inversion model.

**Section S1.**

As the first step after estimating the MT responses, dimensional analysis of the dataset has been performed to investigate the Z tensor properties, which reflect the symmetries of the subsoil structures and particularly their privileged alignments. For such an analysis, the phase tensor (PT) introduced by Caldwell et al. (2004) ^65^ has been derived directly from the Z tensor through the relationship PT=Re(Z)^-1^·Im(Z). The PT is insusceptible to galvanic distortions and can be represented graphically as an ellipse with the principal axes (Φ_max_ and Φ_min_) showing the major-minor axes of the tensor. The polarization ellipses behaviour, estimated through the well-known MTpy code^66,67^, has been reported for a few selected periods in Fig. SM02. Such ellipses can be employed to estimate which parts of the impedance tensor could be considered 1D, 2D or 3D, once the ellipticity (λ) and, in the case of nonsymmetric, the skew-angle (β) have been defined as a function of Φ_max_ and Φ_min_ ^68^. For every single period, the dimensionality of the MT dataset can be evaluated according to the criterion 1D: λ ≈ 0, β ≈ 0; 2D: λ ≠ 0, β ≈ 0, (α-β) constant; 3D: λ ≠ 0, β ≈ 0 or β ≠ 0, but (α-β) no constant^69^. A threshold value has been considered for the skew angle β (5 degrees) and the ellipse's eccentricity λ (0.1). Data dimensionality for each frequency and each measurement site is represented by an array with values of 1, 2 or 3, visually sketched in Figure SM03. Such a PT analysis confirms the 3D character of the central sector of the Campi Flegrei Caldera.

The second step consists of analyzing the influence of the ground-level topography and seafloor bathymetry on the MT dataset. A sea layer around the survey area severely affects the observed MT responses due to the sharp electrical contrast between seawater and land. The skin depth of the performed MT survey can reach up to a few kilometres, so the sea likely has a substantial influence on observed MT data when the separation distance from the coast is smaller than the skin depth of the frequency of interest ^70^, which is likely the case of the presented survey. Additionally, the undulating topographic features modify the current flow pattern, thus affecting the electrical and magnetic field components to different degrees; in observation sites located in this vicinity, the MT response function may become distorted ^71^. A forward modelling study permitted the evaluation of the frequencies most subject to the influence of these two effects and whether these could disturb the diagonal and nondiagonal modes of the **Z** tensor in the same manner. Such forward modelling reconstructed the MT response of a theoretical model of the CFc, which considers the presence of the real ground-level topography and seafloor bathymetry over an ideal homogeneous half-space of 100 Ωm resistivity. The forward modelling, realized by adopting the ModEM 3D code^59^ and the same model mesh adopted for the inversion procedure (described in the main text), permitted the reconstruction of the corresponding synthetic behaviour of the apparent resistivity ρ (log10(Ωm)) and phase Φ (degrees). The simulation evaluates how much the MT responses of the tested model remain substantially unaffected by topography- and bathymetry-related distortion with respect to the one that should characterize the ideal homogeneous half space (e. g. ρ=100 Ωm and Φ=45°). The results are summarized in Fig. SM04, where apparent resistivity maps have been reproduced for all the 4 modes of the Z tensor, in correspondence with four different periods. Looking at those maps, it can be assessed that the nondiagonal modes remain substantially unaffected by topography- and bathymetry-related distortion, with minor distortions below 1 Hz. On the other hand, considering the diagonal modes, the most severe perturbation affects the data over all the investigated frequencies. These results have been considered during the inversion phase when the choice of error floors imposed on the data has been made.

**Section S2.**

Once the preferred inversion model had been selected, we tried to evaluate the effects on the inversion results if 2D approximation was performed. Synthetic MT curves of apparent resistivity and phases have been generated in correspondence with an ideal rectilinear transect. The adopted ModEM forward code has a 3D nature. In such a way, the influences of the three-dimensional character of the preferred inversion model reflect in the synthetic data. The ideal transect alignment is similar to the directions of the transects presented by Troiano et al. (2014) and Siniscalchi et al. (2019). Adopting the 2D commercial code WingLink^®^, we performed a 2D inversion of the synthetic dataset. After adding 5% Gaussian noise, the apparent resistivity and phase curves related to the nondiagonal modes of the impedance tensor were inverted. By comparing the results of the WingLink inversion with the resistivity cross-section extracted from the preferred resistivity model along the ideal transect, we evaluate the spurious effects of 2D inversion (Fig. SM05).

**Section S3.**

*Resolution tests.*

The consistency of the main features of the preferred inversion model was questioned by performing a series of tests in order to analyze every significant structure separately.

First, we adopted the MTpy code to evaluate the penetration depths of the electromagnetic waves at a 3 s period, using the Niblett-Bostick transform (Kirkby et al., 2019 and references therein), which map is reported in Figure SM06. In the second step, we perform synthetic tests on the data sensitivity of certain model parts. Due to the complexity of 3D, the model perturbation method has been used for this. The resistivity for different anomalies in the preferred inversion model has been modified, decreasing and increasing its value in a range uniformly covering several magnitude orders. Then, many forward modelling runs were launched, one for each resistivity value tested, and the difference between the new nRMS and the one related to the inversion preferred model (about 1.7; see Fig. SM07) was checked. If the difference is significant, data are sensitive enough to that part of the model. After checking all the critical anomalies in the 3D model by perturbation, it is possible to know which parts of the model are well-constrained by the data and more reliable than others.

This analysis was performed by questioning the following anomalies:

1. the three C1-C2-C4 conductive anomalies;

2. the deeper part of the S-PHS;

3. the R3 resistive anomalies;

4. the R4 resistive anomalies;

The results of the resolution tests are resumed in Figure SM08. The nRMS variation retrieved testing the resolution of the C1-C2-C4 conductive formation is plotted in Figure SM08a as a function of the modified resistivity attributed to the block. The nRMS changes between 5.2 and 23.78 as the resistivity of the area progressively increases. In other words, the nRMS changes range between 180% and 1300% with respect to the nRMS related to the preferred inversion model. Such a level of a modification indicates that the C1-C2-C4 structures are well resolved. Changes in deeper zones induce the lowest changes in nRMS, as evident when the sensitivity analysis for the R3 and R4 resistive formations, summarized in Figure SM08c,d, are considered. In such cases, the change in nRMS varies between 2.62 and 6.05, e.g. nRMS changes between 50% and 300% with respect to the nRMS related to the inversion preferred model. The shape of the curve confirms that the more convincing hypothesis for these structures consists of highly resistive bodies, considering that the nRMS rapidly increases when more conductive formations are considered. Figure SM08b shows the results obtained testing the resolution of the bottom of the S-PHS. In this case, the retrieved nRMS vary between 2.68 and 2.88, e.g. between 55% and 70% with respect to the inversion preferred model.

To quantify whether or not the edited resistivity model statistically differs from the preferred inversion model, we adopted the Kolmogorov–Smirnov (K–S) test, applied using the kstest2 Matlab routine. The K-S is a non-parametric hypothesis test that determines whether two populations are from the same continuous distribution within some significance level^31^. As two populations to be tested, we adopted the residuals (normalized by error) from the preferred inversion model and the residuals (normalized by error) of the calculated response of the edited model. If the null hypothesis is accepted (H=0; p-value greater than significance level), then we cannot conclusively determine if the two sets of residuals are drawn from the same or different distributions. On the other hand, if the null hypothesis is rejected (H=1; p-value less than significance level), we conclude that the two sets of residuals are distinct, and thus the added anomaly has a statistically significant impact on the inversion response. In other terms, we will consider the anomaly in the edited model as 'detected' by the MT data if the K–S tests return a statistically significant p-value (null hypothesis is rejected; H=1).

In the following, Table 1 reports the H (Table 1; second column) and p (Table 1; third column) values, which result from applying the K-S test. Also, the nRMS values have been reported for the estimated response of the edited models with respect to the measured data.

| id | ρ [Ωm] | H | p | nRMS |
| --- | --- | --- | --- | --- |
|  |  |  |  |  |
| C124 | 0.1 | 1 | 1.74E-29 | 5.2 |
| C124 | 1 | 1 | 3.7E-13 | 4.74 |
| C124 | 10 | 1 | 4.30E-12 | 10.04 |
| C124 | 100 | 1 | 1.5E-25 | 17.17 |
| C124 | 1000 | 1 | 8.2E-33 | 21.79 |
| C124 | 10000 | 1 | 9.89E-34 | 23.78 |
|  |  |  |  |  |
| R3 | 0.1 | 1 | 4.0E-09 | 6.05 |
| R3 | 1 | 1 | 6.9E-07 | 6.51 |
| R3 | 10 | 1 | 4.6E-04 | 6.25 |
| R3 | 100 | 1 | 0.059 | 4.05 |
| R3 | 1000 | 1 | 0.05 | 2.7 |
| R3 | 10000 | 0 | 0.06 | 2.63 |
|  |  |  |  |  |
| R4 | 0.1 |  |  | 4 |
| R4 | 1 | 1 | 3.5E-11 | 3.57 |
| R4 | 10 | 1 | 4.7E-07 | 3.26 |
| R4 | 100 | 1 | 0.01 | 2.78 |
| R4 | 1000 | 1 | 0.05 | 2.61 |
| R4 | 10000 | 0 | 0.06 | 2.38 |
|  |  |  |  |  |
| F | 0.1 | 1 | 0.03 | 2.88 |
| F | 1 | 1 | 0.05 | 2.7 |
| F | 10 | 0 | 0.12 | 2.62 |
| F | 100 | 0 | 0.1 | 2.62 |
| F | 1000 | 1 | 0.06 | 2.75 |
| F | 10000 | 1 | 0.04 | 2.85 |

The results resumed in Table 1 confirm that conductive C1-C2-C4 structures are well resolved. Similarly, the performed resolution tests also confirm the resistive nature of the R3 and R4 structures. To further support the conductive behaviour of the lower part of the S-PHS (F), which is the anomaly of the preferred inversion model less constrained by the data, we reported in Fig. SM09 a comparison between two of the tested edited models (F_0.1_ and F_100_, where the resistivity has been edited to 0.1 Ωm and 100 Ωm, respectively), each one plotted as a function of the residual of the preferred inversion model. The F_100_ is the most similar to the preferred inversion model among the two edited models. Consequently, its residuals appear less widely scattered with respect to the residuals of the F_0.1_ model.

*Supplementary Figure captions.*

Fig. SM01. Apparent resistivity (log10(ρ) in Ωm) and phases (φ in degrees) relative to the four modes of the impedance tensor **Z** for the MT soundings MT1, MT9 and MT20 (see Figure 1 for site locations).

Fig. SM02. Maps of the Phase Tensor ellipses. The inner colour of the ellipses indicates the value of the Skew angle β (in degrees) (see [55]). The panels respectively refer to 0.001s, 0.01 s, 0.1 s and 1 s.

Fig. SM03. Summary of the data dimensionality for each frequency and each measurement site. The figure represents which parts of the impedance tensor could be considered 1D, 2D or 3D based on the results of the Phase Tensor analysis performed using the MTpy code. Threshold values of 5° for the skew angle β and 0.1 for the eccentricity of the ellipses have been adopted.

Fig. SM04. Results of the forward model study aimed to reconstruct the effects of the ground-level topography and seafloor bathymetry on the MT dataset. The actual coastline and undulating topography of the CFc central sector have been superimposed over a 100 Ωm homogeneous half-space. The corresponding synthetic apparent resistivity (log10(Ωm)) maps (related to each one of the four Z tensor modes) have been evaluated using the ModEM code and subsequently represented for periods of 0.001 s, 0.01 s, 0.1 s and 1 s, respectively.

Fig. SM05. Results of forward modelling aimed at evaluating the likely presence of distortion effects in the case of 2D inversion of the MT data. A synthetic dataset has been calculated adopting the 3D code ModEM, the preferred inversion model, and an ideal straight transect, which direction is shown in panel a). Blue dots indicate the locations of the ideal measurement sites, which replicate the one presented by Siniscalchi et al. (2019). The resistivity cross-section extracted by the preferred inversion model along the ideal 2D transect is reported in panel b). The resistivity section obtained through 2D inversion of the synthetic dataset has been reported in panel c).

Fig. SM06. Map of the penetration depth of the electromagnetic waves of 3 s period, obtained using the Niblett-Bostick transform.

Fig. SM07. Map of the nRMS misfit values. A total nRMS of 1.7 was achieved.

Fig. SM08. Results of resolution tests on the most significant anomalies detected by the preferred inversion model. The panels report the nRMS changes (on the y-axis) vs resistivity imposed in specific regions of the preferred inversion model (on the x-axis). The tested zones are (C124) the conductive C1-C2-C4 anomalies; (F) the lower part of the S-PHS; (R3) the R3, and (R4) the R4 resistive anomalies.

Fig. SM09. (a) plot of the residuals for the edited model F_0.1_ vs the residual of the preferred inversion model. (b) plot of the residuals of the edited model F_100_ vs the residual of the preferred inversion model.
